# Subwavelength pixelated CMOS color sensors based on anti-Hermitian metasurface

**DOI:** 10.1038/s41467-020-17743-y

**Published:** 2020-08-06

**Authors:** Joseph S. T. Smalley, Xuexin Ren, Jeong Yub Lee, Woong Ko, Won-Jae Joo, Hongkyu Park, Sui Yang, Yuan Wang, Chang Seung Lee, Hyuck Choo, Sungwoo Hwang, Xiang Zhang

**Affiliations:** 1 Nano-Scale Science and Engineering Center (NSEC), 3112 Etcheverry Hall, University of California Berkeley, Berkeley, CA 94720 USA; 2 Samsung Advanced Institute of Technology, Samsung Electronics Co. Ltd., Samsung-ro 130, Yeongtong-gu, Suwon-si, Gyeonggi-do, 16678 Korea; 3 Faculties of Sciences and Engineering, University of Hong Kong, Hong Kong, China

**Keywords:** Optoelectronic devices and components, Sub-wavelength optics, Imaging and sensing

## Abstract

The demand for essential pixel components with ever-decreasing size and enhanced performance is central to current optoelectronic applications, including imaging, sensing, photovoltaics and communications. The size of the pixels, however, are severely limited by the fundamental constraints of lightwave diffraction. Current development using transmissive filters and planar absorbing layers can shrink the pixel size, yet there are two major issues, optical and electrical crosstalk, that need to be addressed when the pixel dimension approaches wavelength scale. All these fundamental constraints preclude the continual reduction of pixel dimensions and enhanced performance. Here we demonstrate subwavelength scale color pixels in a CMOS compatible platform based on anti-Hermitian metasurfaces. In stark contrast to conventional pixels, spectral filtering is achieved through structural color rather than transmissive filters leading to simultaneously high color purity and quantum efficiency. As a result, this subwavelength anti-Hermitian metasurface sensor, over 28,000 pixels, is able to sort three colors over a 100 nm bandwidth in the visible regime, independently of the polarization of normally-incident light. Furthermore, the quantum yield approaches that of commercial silicon photodiodes, with a responsivity exceeding 0.25 A/W for each channel. Our demonstration opens a new door to sub-wavelength pixelated CMOS sensors and promises future high-performance optoelectronic systems.

## Abstract (web summary)

Pixel size in imaging and displays is limited by fundamental constraints that compromise performance at wavelength scales. Here the authors present subwavelength color pixel sensors based on anti-Hermitian metasurfaces relying on structural color for increased performance.

## Introduction

Resonant systems are critical to pixelated image sensors, which can find a broad range of applications in photovoltaics, imaging, and display systems. Conventional resonators are largely constrained by the fundamental diffraction limit and absorb a broad part of the spectrum with considerable overlap, leading to low spectral and imaging resolution. It becomes increasingly clear that all imaging and display systems suffer from performance issues when their dimensions approach the wavelength of absorbed light. Two outstanding problems with conventional pixels may be classified as optical crosstalk and electrical crosstalk due to unwanted light scattering and diffusion of charge carriers to neighboring pixels^1–3^.

Anti-Hermitian (AH) systems have recently gained increasing attention due to their unique ability to increase the selectivity of resonant devices^4,5^, which are uniquely positioned to address both optical and electrical crosstalk, enabling pixels with sub-wavelength dimensions, promising for future optoelectronic systems. At the AH-coupling condition, the absorption profiles become sharpened to the extent that the overlap in absorption spectra, also known as crosstalk, becomes negligible^4^. The increase in quality factor of the resonator due to destructive interference between incoming and outgoing waves, leads to designed reflection minima and absorption maxima at the desired spectral location. Till now, AH systems were mostly studied in the fields of quantum optics and nuclear physics, with other crosstalk mitigation schemes proposed for all-dielectric systems^6–8^. Anti-Hermitian coupling has been recently employed in micro- and nanophotonics with potential technological applications in photovoltaics, imaging, and display systems^4,5,9–12^. However, current state-of-the-art AH systems have been restricted to a single spatial dimension, limited color channels, and lacked electrical integration with complementary metal-oxide-semiconductor (CMOS) compatible materials, which has hindered their adoption in practical settings.

In this work, we report the first CMOS compatible, two-dimensional (2D), AH metasurface based color image sensors. The AH silicon metasurfaces with two-dimensional arrays of three differently sized nanocylinders are coupled with a shallow p-i-n junction for efficient carrier transport and electrical readout. By carefully controlling the size and separation of silicon (Si) nanocylinders, visible light can be selectively absorbed in multiple color channels with negligible diffraction, leading to spectrally pure absorption profiles of neighboring sub-pixels. In addition, due to their three-dimensional, non-planar morphology, nanocylinders composed of shallow p-i-n junctions are inherently immune to electrical crosstalk as charge carriers generated in the intrinsic region are separated by air from the intrinsic region of neighboring sub-pixels. In addition to being the first demonstration of an AH system with p-i-n junction, three visible channels, and two-dimensional configuration, this sub-wavelength anti-Hermitian metasurface sensor covers 28,000 pixels, which is able to sort three colors over a 100 nm bandwidth in the visible regime, independently of the polarization of normally incident light. The responsivity of the AH devices is the same order of magnitude as commercial silicon photodiodes, exceeding 0.25 A/W for all colors., which presents a great practical value for the imaging and display industries, where solutions to the small pixel problem are needed.

## Results

### Design of CMOS anti-Hermitian metasurface

**Fig. 1:**
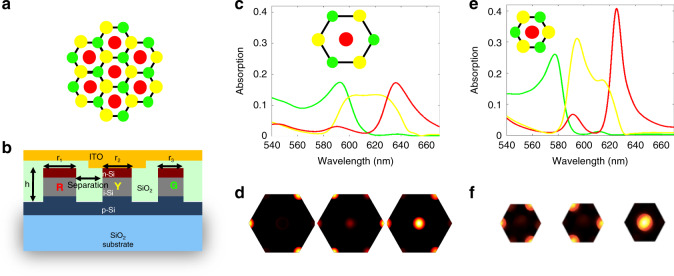
Optical design and simulation of three-channel, two-dimensional, anti-Hermitian PIN Si metasurface. **a** Top- and **b** side-view schematic of 3-color, 2-D array of silicon nanocylinders. **c**, **e** Simulated absorption spectra of normally incident light in green-, yellow-, and red-absorbing nanocylinders for lattice constants of **c** 350 nm, mixed coupling condition, and **e** 220 nm, anti-Hermitian coupling condition. **d**, **f** Simulated dissipated power in each nanocylinder at the associated wavelength of peak absorption (590, 610, and 640 nm in **d** and 575, 590, and 625 nm in **f**). It can be seen that when the lattice constant is above the anti-Hermitian regime, the three-color spectra are broad and overlapping, leading to optical color crosstalk between three-color channels. The optimal structure designed by the principle of anti-Hermitian coupling has enhanced quality factors, leading to a significant reduction of color crosstalk and improvement of the peak absorption efficiency. Source data for **c** and **e** are available in the Source Data File tabs labeled **1c** and **e**, respectively.

The color-sorting metasurface is composed of three types of silicon nanocylinders with different diameters *d*, patterned into a hexagonal lattice with a sub-wavelength center-to-center nanocylinder spacing, *a*, of 220 nm, as illustrated in Fig. 1a. From Mie theory, silicon nanoparticles can support strong magnetic dipole resonances in the visible spectral range^13–16^. Here, the sizes of the three types of nanocylinders are optimized to achieve selective absorption in the red (*d*_R_ = 136 nm), yellow (*d*_Y_ = 122 nm) and green (*d*_G_ = 104 nm) part of the visible spectrum, respectively (herein denoted [*d*_R_*d*_Y_*d*_G_] = [136 122 104] nm). The silicon nanocylinders are doped into vertical p-i-n junctions, and a thin transparent layer of indium tin oxide (ITO) is sputtered on top as electrical contact, shown schematically in Fig. 1b. The neighboring nanocylinders are electrically isolated from one another by the air gap between the intrinsic and n-doped regions, resulting in negligible electrical crosstalk.

While the optical coupling coefficient *κ*_*ij*_ is generally a complex number, the AH condition is achieved by carefully choosing the separation distance between nanocylinders, such that the real part from the direct and indirect coupling cancel each other, resulting in sharpened resonances and reduced optical crosstalk (Supplementary Note 1). The real and imaginary coupling constants between yellow-absorbing and green-absorbing nanocylinders, and yellow-absorbing and red-absorbing nanocylinders, are shown in Supplementary Fig. 1a–f, respectively, as a function of the center-to-center separation distance, *a*, for three different scaling factors of the nanocylinder diameters.

When *a* = 350 nm, far from the AH condition, broad, overlapping absorption spectra are observed, which leads to relatively low peak absorption values as well as color crosstalk if these nanocylinders were to be used as pixels (Fig. 1c, d). By contrast, Fig. 1e, f shows the simulated absorption spectra and dissipated power of the same nanocylinders with *a* = 220 nm, which corresponds to an optimized AH condition with consideration of fabrication constraints. It can be clearly seen that selective chromatic absorption is achieved at three distinct colors with small overlapping. The peak absorption efficiencies reach 30%, owing to the large absorption cross-section of the silicon nanocylinders. Because the derivate of the coupling constants with respect to the separation distance is relatively small (Supplementary Fig. 1), small deviations as ‘near-anti-Hermitian’ from the optimal geometry are expected to also yield good performance in terms of peak absorption and low crosstalk. Simulation results of metasurfaces with varying scale factors, shown in Supplementary Fig. 2, verify this expectation, thus permitting deviations from ideal designs based on constraints associated with fabrication of the nanocylinder array.  After being optimized for their optical response, simulations of photocurrent generation in the nanocylinders, shown in Supplementary Fig. 3, confirmed their viability for efficient photodiode operation. 

### Fabrication of CMOS anti-Hermitian metasurface

**Fig. 2:**
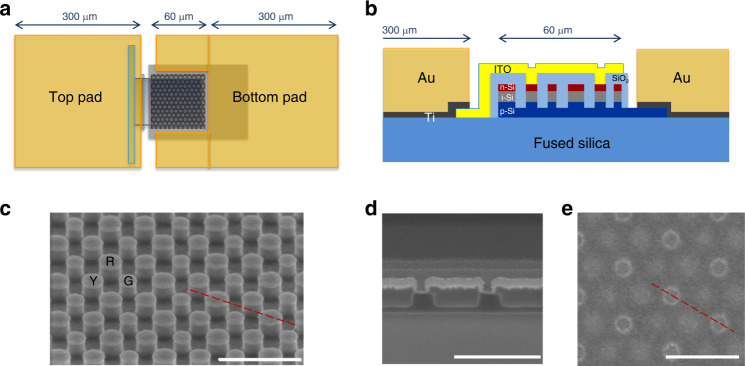
Device layout of CMOS color sensors based on anti-Hermitian metasurfaces. **a** Top- and **b** side-view of color sensor device layout including electrical contacts. **c** Oblique view of scanning electron micrograph, just after nano patterning of PIN Si rods. **d** Side cross-section view and **e** top view of nanocylinder array, after SiO_2_ gap-filling and ITO electrode deposition. The white scale bar corresponds to 500 nm in **c**–**e**. The diagonal red line in **c** and **e** indicates the cross-section presented in **d**.

Conversion of optical energy into electrical photocurrents with minimal crosstalk requires well-controlled fabrication of 130-nm-thick poly-Si layer vertical p-i-n junction and accurate control of nanocylinders’ diameter and center-to-center distance in a hexagonal lattice.  A schematic of the complete device is shown in Fig. 2. The p-i-n junction prepared in this work is successively laminated, forming p-type Si, intrinsic Si and n-type Si with thickness of 50–70 nm, 30–70 nm, and 10–30 nm, respectively. Contrary to previous research of color sorting using 2.7-μm-thick PIN Si rods^17^, AH-coupling design of 0.22 μm sub-pixels faces the challenge to create a vertical p-i-n junction of Si with a thin thickness of just 130 nm. Supplementary Figure 4 summarizes the process steps for creating the vertical shallow junction PIN Si layer devised in this study. Extinction coefficient (*k*) of PIN poly-Si is measured as a low value of 0.097 as well as high refractive index of 4.269 at 550 nm wavelength. Poly-Si deposition and ion implantation are applied to forming vertical p-i-n junction as a mass productive method. The complete fabrication process is summarized in “Methods” section and Supplementary Fig. 5.

According to the design of the AH metasurface, the optimized diameters of the standard scale nanocylinders are 104, 122, and 136 nm, which correspond to green, yellow, and red sub-pixels, each with the height of 100 nm. ITO electrode, connected to Au pad (top pad), has contact with one n-type Si of three colors. P-type Si acts as an electrical common ground and is connected to another Au pad (bottom pad), as shown in Fig. 2b. 30 nm-thick SiO_2_ separates the upper regions of n-type Si from ITO electrode (see Supplementary Note 2 for discussion on blue part of spectrum).

**Fig. 3:**
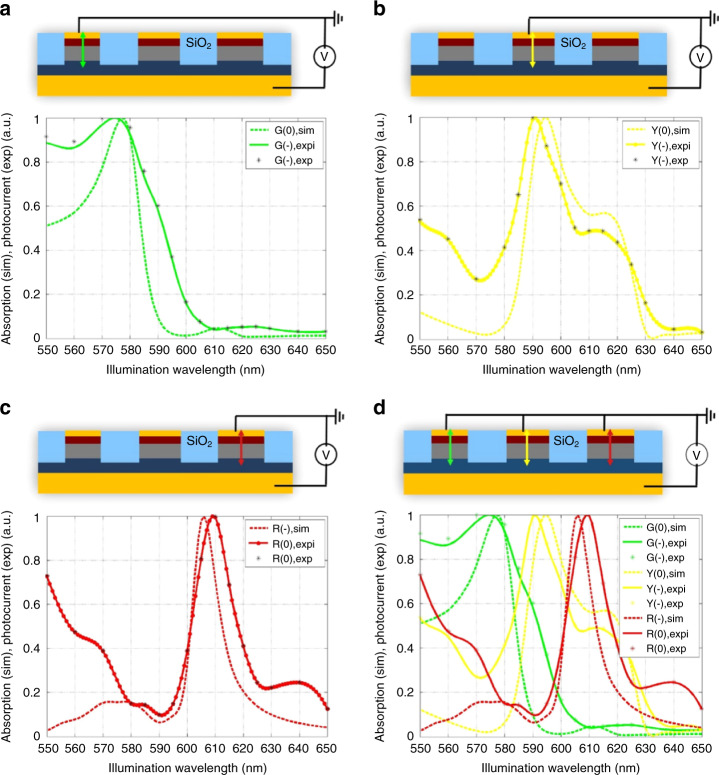
Experimental three-channel color-sorting in anti-Hermitian PIN Si metasurface. Experimental photocurrent (solid curves) and simulated absorption (dashed curves) spectra of the AH PIN Si metasurface when electrical connection is made to **a** green, **b** yellow, and **c** red color channels. For clarity, all curves are normalized to their peak values. **d** Combined photocurrent and absorption spectra of all three channels. Legend labels “sim”, “expi”, and “exp” refer to simulated, interpolated experimental, and raw experimental data, respectively. Source data for **a**–**d** are provided in the Source Data File tabs labeled **a**–**c**, respectively.

### Experimental demonstration of high-performance color-sensing

Devices designed for AH coupling were compared to planar devices with identical PIN silicon material makeup but lacking nanostructure via etching. The device clearly exhibits diode-behavior with large forward-bias currents and small reverse-bias currents (Supplementary Fig. 6). Consistent with photodiode behavior, as the illumination power increases, the current increases for a fixed reverse-bias level, indicating that the devices show the necessary behavior for color and image sensors^18^. To separate the optical response of the AH devices from their electrical transport properties, reflection spectra were measured of control devices and AH devices of varying scale. Whereas the control devices exhibit a single reflection dip, as shown in Supplementary Fig. 7, each AH metasurface shows three distinct reflection minima, which are associated with absorption maxima, as shown in Supplementary Fig. 8.

**Fig. 4:**
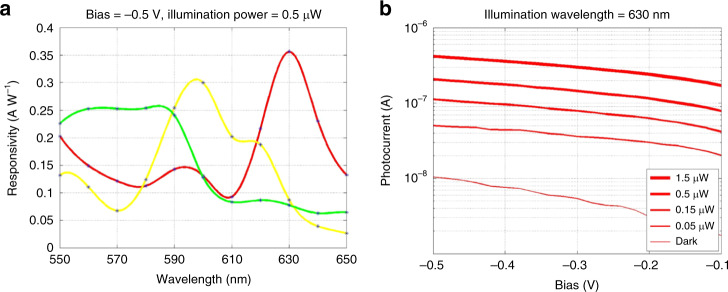
Performance characteristics of CMOS color sensors based on anti-Hermitian metasurfaces. **a** Measured responsivity as a function of wavelength at reverse-bias of −0.5 V and illumination power of 0.5 μW for green, yellow, and red color channels. **b** Measured photocurrent as a function of reverse-bias in red color channel at illumination wavelength of 630 nm with illumination power parameterized. Source data for **a** and **b** are provided in the Source Data File tabs labeled **a** and **b**, respectively.

With these superior optical behavior, the combined optical and electrical response was probed via photocurrent spectroscopy. Figure 3a–d shows the normalized photocurrent spectra of individually addressed devices designed to absorb red, yellow, and green light, along with the normalized simulated absorption spectra. Sharp resonances with peak wavelength and quality factors comparable to those predicted by numerical simulations are observed. Figure 4a shows the measured responsivity, defined as the ratio of photocurrent to illumination power, of the three-color channels, with values of approximately 0.35 A/W, 0.30 A/W, and 0.25 A/W for red, yellow, and green, respectively, at the bias voltage of −0.5 V and illumination power of 0.5 μW. Fixing the wavelength to 630 nm, Fig. 4b shows increasing photocurrent with illumination power, which is approximately linear over this range, indicative of proper photodiode behavior. By contrast, the photocurrent spectra of the control devices lacked any resonant character and samples with poorly designed nanocylinder spacing exhibited overlapping resonances, with significant optical crosstalk. Based on these results we confirm the observation of near-AH coupling with high responsivity in 3 visible channels in the shallow junction PIN silicon metasurface. Furthermore, photocurrent measurements of metasurfaces with varying scale factors demonstrate that the AH condition is robust to small geometric changes, as shown in Supplementary Fig. 9.

## Discussion

**Fig. 5:**
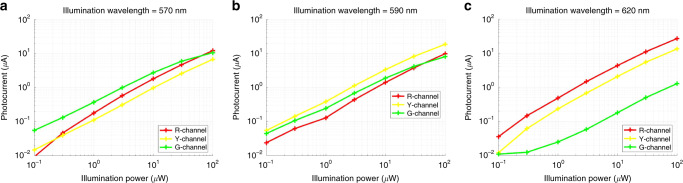
Measured photocurrent as a function of illumination power. Measured photocurrent in green, yellow, and red  color channels as a function of illumination power at illumination wavelengths of **a** 570 nm, **b**, 590 nm, and **c** 620 nm. Color-sorting behavior is consistently observed over three orders of magnitude of illumination power. Source data for **a**–**c** are provided in the Source Data File tabs labeled **a**–**c**, respectively.

In addition to responsivity, the performance of photodiodes, generally, and CMOS color sensors, specifically, is often evaluated in terms of the dependence of the photocurrent on illumination power^18^. To the best of our knowledge, an analysis of anti-Hermitian color-sorting performance with respect to illumination power has yet to be reported. Figure 5 displays the measured photocurrent as a function of illumination power for the red, yellow, and green color channels at three different illumination wavelengths, representative of the resonant wavelengths of each channel. High responsivity color sorting is clearly observed over nearly three orders of magnitude for each illumination wavelength. Aside from saturation effects appearing at the highest power level for the green channel at the green wavelength, the green, yellow, and red channels absorb at least twice the power of the other channels at the green, yellow, and red illumination wavelengths, respectively.

Lastly, the dependence of the device’s performance on the polarization and angle of the incident light is also of practical importance. We found that for normal incidence, the absorption spectrum presents a very good isotropic behavior and does not depend on the incident light polarization (Supplementary Fig. 10a). When the incidence is angled, the absorption spectrum is different for TE mode incidence (electric field parallel to the metasurface) and TM mode incidence (magnetic field parallel to the metasurface). For TE mode incidence, the color image sensor can accept a large incident angle up to ±30° and still has acceptable color-sorting ability (Supplementary Fig. 10b). For TM mode incidence, however, the device can only tolerate ±5° incident angle with current design. This is because when the incident electric field has a component perpendicular to the metasurface, the excited optical modes become different, which leads to a significant change in their coupling coefficient.

The insensitivity of color-sorting behavior with respect to illumination power, along with the high responsivity, make CMOS color sensors based on anti-Hermitian metasurfaces with sub-wavelength pixels a compelling subject for future research and development. Future directions for this work include integration with CMOS readout circuit arrays (Supplementary Fig. 11), extension of the spectral response to the blue end of the visible spectrum and optimization of the geometry to provide color-sorting for obliquely angled excitation.

As the demand for smaller pixel size and higher resolution in imaging and display technologies increases, our work advances the state-of-the-art by showing for the first time, PIN readout, three-color sorting over a two-dimensional surface without sacrificing responsivity. Furthermore, the sub-wavelength sized pixels are demonstrated based on the principle of AH coupling and fabricated via CMOS-compatible processes into vertical shallow junction PIN nanocylinders that efficiently convert optical energy to a clear electrical readout without crosstalk. Our work promises future compact, small pixelated, high-performance optoelectronic systems.

## Methods

### Numerical simulation

The optical properties of the silicon metasurface were simulated using fully 3-D finite-element method (Comsol Multiphysics 5.0). The refractive indices of the doped and intrinsic silicon used in the simulation came from ellipsometry measurement of the actual materials. Absorption spectra were simulated as a function of nanocylinder height, radius, and separation, with the dependence of nanocylinder resonant frequencies and quality factors being the object of study. Optimal values were found that minimized the real part of the coupling constant and maximized absorption at three desired wavelengths, representative of red, yellow, and green colors. The coupling constant is derived from the simulated eigen frequencies of the isolated nanocylinders and the coupled lattices. The coupling constants correspond to the difference between the eigen frequencies of resonators in isolation versus resonators in the presence of neighboring resonators. For example, denoting the complex resonance frequency of the individual red pixel lattice and yellow pixel lattice as *ω*_R_ and *ω*_Y_, respectively, and the complex eigenfrequency of the hybrid mode (with the red and yellow pixel lattices both present) as *ω*_+_ and *ω*_−_, the coupling constant between the red and yellow channels can be calculated as:

$$\kappa = \frac{1}{2}\sqrt {(\omega _ + - \omega _ - )^2 - (\omega _{\mathrm{R}} - \omega _{\mathrm{Y}})^2}.$$

Because the eigen frequencies are complex-valued, the difference between them has a real and imaginary part. Simulations of electrical transport were conducted by simultaneously solving the Poisson and drift-diffusion equations with source fields from optical absorption simulations using finite-element method (Lumerical DEVICE).

### Device characterization

IV measurements were conducted using a source meter (Keithley 2400) that both supplied voltage and measured current under dark and illuminating conditions. The samples were wire bonded (WestBond) to a carrier chip (Addison Engineering) and placed in a breadboard. Standard BNC cables and leads were used to connect the source meter to the breadboard. Photocurrent measurements were conducted with the same source meter in conjunction with a narrowband tunable light source (Coherent Chameleon Compact OPO Vis), enabling detection without the need for a lock-in amplifier. The light source illumination intensity was controlled with a variable neutral density attenuator and power meter (Newport 843R) with a silicon photodiode receiver (Newport 918D-SL-OD3R). Sample alignment was achieved via low numerical aperture (NA) microscope objective and camera (ThorLabs). Responsivity was calculated as the ratio of measured photocurrent, with dark current subtracted, to the optical power incident on the active area of the device. Measurements with light focused using both low (NA = 0.1) and high (NA = 0.6) NA objectives were conducted. When accounting for the different beam waists, comparable responsivity values were observed. Prior to measuring photocurrent of the control or anti-Hermitain devices, responsivity of commercially manufactured silicon photodiodes (Vishay Semiconductors VBP104S) was measured and matched to its specifications as a means of qualifying the measurement setup. Reflection spectra were measured using a broadband white light source (Newport HAL-1000) and liquid nitrogen-cooled spectrometer (Princeton Instruments Spectra Pro 2300i) with the light focused with a high NA objective onto the active area of the devices.

### Sample fabrication

Low pressure chemical vapor deposition (LPCVD) is used to deposit poly-Si on a fused silica substrate under SiH_4_ flow at 60sccm and a pressure of 150 mTorr. A 50-nm-thick SiO_2_ is added on the poly-Si layer by PECVD at 250 °C. SiH_4_ and N_2_O gas at flow rates of 20 and 1800 sccm respectively, for forming the capping SiO_2_ thin films. After ion implanting at the energy of 3–10 keV, the capping layer removed by etching with an aqueous mixture of hydrofluoric acid and ammonium fluoride (2.35% HF + 17% ((NH_4_)F) + deionized water). Nano cylinder arrays with diameters of 100 nm were defined by an e-beam lithography system (JBX-9500FS, JEOL Co., Japan). A positive e-beam resist (ZEP520A, Zeon, Japan) is spun on a PIN poly-Si film. The resist is developed in a 2.38% tetramethylammonium hydroxide (TMAH) solution. Using the e-beam resist’s pattern as an etching block mask, nanocylinder arrays are fabricated. A gas mixture of C_4_F_8_, SF_6_, and Ar with flow rates of 45, 39, and 10 sccm respectively, is used at a chamber pressure of 10 mTorr. A conformal SiO_2_ deposition with O_2_ and TEOS source is applied to narrow-gap filling, having power of 350 W, gas flow rates of 220 sccm, pressure of 7 Torr and working temperature of 400 °C. An RIE trimming with Ar plasma is assisted for void-free nano-gap filling, under a flow rate of 60 sccm and RF power of 300 W and pressure of 10 mTorr.

Diffusivities of several dopants (i.e., boron for p-type doping, phosphorus and arsenic for n-type doping) are considered for constructing thin layer of vertical PIN Si on the basis of previous studies^19–22^. Thermal diffusion of boron from underneath oxide is used to restrict p-type Si thickness and prevent native oxide from forming at the interface between p-type Si and intrinsic Si. Boron ions are implanted on the surface such as SiO_2_ or transparent conductive oxide (TCO) in advance, then poly-Si layer is deposited. Through rapid thermal processing (RTP) at 1000 °C, boron dopants diffuse into poly-Si thin film and they are activated as acceptors of p-type Si. The penetration depth of boron elements is determined by RTP durations (e.g., 10 and 30 s durations correspond to 50 and 70 nm depth, respectively).

N-type Si is formed by implanting arsenic ions directly on the top of poly-Si layer. Before arsenic ion implanting, SiO_2_ capping is coated on poly-Si. The penetration depth of arsenic elements depends on the thickness of capping layer and ion implantation energy. The thickness of n-type Si varies by controlling implantation energies in these experiments, as SiO_2_ capping is fixed 50-nm-thick. The diffusion depths of n-type dopants (phosphorous, arsenic) into poly-Si are compared by experiments in this study, keeping implantation energy of 3 keV and RTP duration of 10 s at 1000 °C. Supplementary Figure S3e shows that phosphorous ions, atomic weight of 30.97, diffuse into all thickness ranges of 130 nm, analyzed by secondary ion mass spectroscopy (SIMS). However, heavier ions of arsenic, atomic weight of 74.92, penetrate into 10–30 nm depth at implantation energies of 3–10 keV. Modifying p-type and n-type Si depths by controlling implantation energy and RTP duration, various thickness of intrinsic Si (30–70 nm) enable to fabricate in the constraint of 130-nm-thick poly-Si as shown in Supplementary Table 1. N-type Si has arsenic concentration of 1 × 10^21^ atoms/cc as donors, which is 20 times higher than boron concentration of 5 × 10^19^ atoms/cc as acceptors in p-type Si. For balancing charge carrier concentration, p-type Si (50–70 nm) is designed thicker than n-type Si (10–30 nm). Figure S3f shows the dopant profiles of SIMS analysis of “i-Si 50” in Supplementary Table 1, which mean the 50 nm thickness of intrinsic Si, having the thickness of 10, 50, 70 nm for n-type, intrinsic, p-type Si, respectively. The complete fabrication process is summarized in Supplementary Fig. S5. Poly-Si nanostructures are defined by inductively coupled plasma (ICP) etching. The vertical sidewalls are an important characteristic for realizing nanocylinders of 100 nm diameter, where C_4_F_8_ gas has a role in side wall passivation during SF_6_ plasma etching^23–25^. After nanoscale patterning via e-beam lithography and ICP etching, a low index material of SiO_2_ (*n* ~ 1.45 at 550 nm) are filled among Si rods. To fill the narrow gap of 90 nm, SiO_2_ is deposited in advance by plasma-enhanced chemical vapor deposition (PECVD) with tetraethyl orthosilicate (TEOS) source, and the protrusion edge of SiO_2_ is removed by physical trimming from Ar plasma in Supplementary Fig. S5e. Afterward, additional SiO_2_ is coated using TEOS source for surface planarization, then etched back until 30–50 nm thickness is left over upper regions of nanocylinder arrays, as shown in Supplementary Fig. S4f–i.

## Supplementary information

Supplementary Information

## Data Availability

The data that support the findings of this study are available from the corresponding author upon reasonable request. All data generated or analyzed during this study are available in this published article. The data presented in the figures of this report are available in the accompanying Source Data file. Source data are provided with this paper.

## Source data

Source Data
