# Supplementary material for: Subwavelength pixelated CMOS color sensors based on anti-Hermitian metasurface
Source: Nat Commun. 2020 Aug 6;11:3916. doi: 10.1038/s41467-020-17743-y (PMC7413260; doi:10.1038/s41467-020-17743-y)
Supplement: Supplementary file 1 — Supplementary Information [file 41467_2020_17743_MOESM1_ESM.pdf]

Supplementary Information

Supplementary Figures 1-11

Supplementary Table 1

Supplementary Notes 1-2

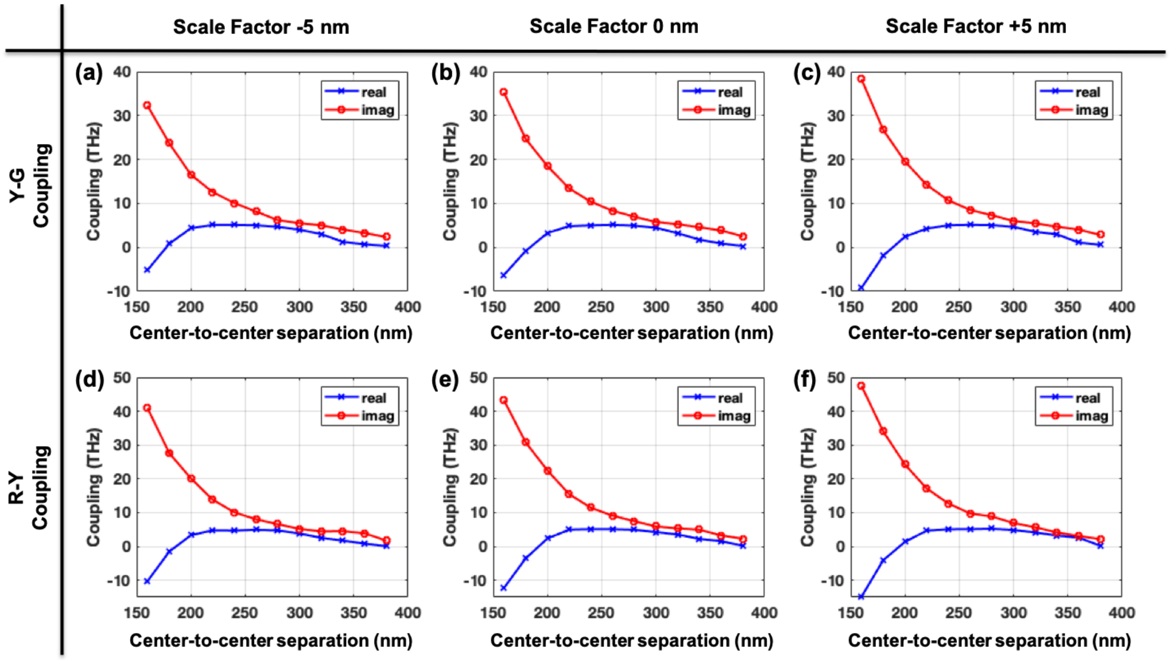

Supplementary Figure 1. Simulated real (blue curves) and imaginary (red curves) coupling constant between two types of silicon nanocylinders as a function of the center-to-center separation distance, denoted as *a*. Coupling constant between (a-c) yellow absorbing and green absorbing nanocylinders, and (d-f) red-absorbing and yellow-absorbing nanocylinders, with scale factors and nanocylinder diameters of (a,d) -5 nm, [131, 119, 97] nm, (b,e) 0 nm, [136, 124, 102] nm and (c,f) +5 nm, [141, 129, 107] nm. When *a* is very small, the coupling is dominated by the direct near-field coupling, which leads to a large negative real part and significant frequency shifting. As *a* increases, the nearfield coupling decreases rapidly, and the real part of the coupling crosses zero, where the direct near-field coupling is canceled by the real part of the far-field coupling, resulting in a purely anti-Hermitian coupling between the nanocylinder sub-lattices. Significantly, it can be noticed that as *a* increases beyond the AH condition, the rate of change of the real and imaginary parts of the coupling becomes smaller. Hence, even near the AH condition, the real coupling is small and imaginary coupling is large, suggesting that near-AH metasurfaces may also perform well as color-sorting devices.

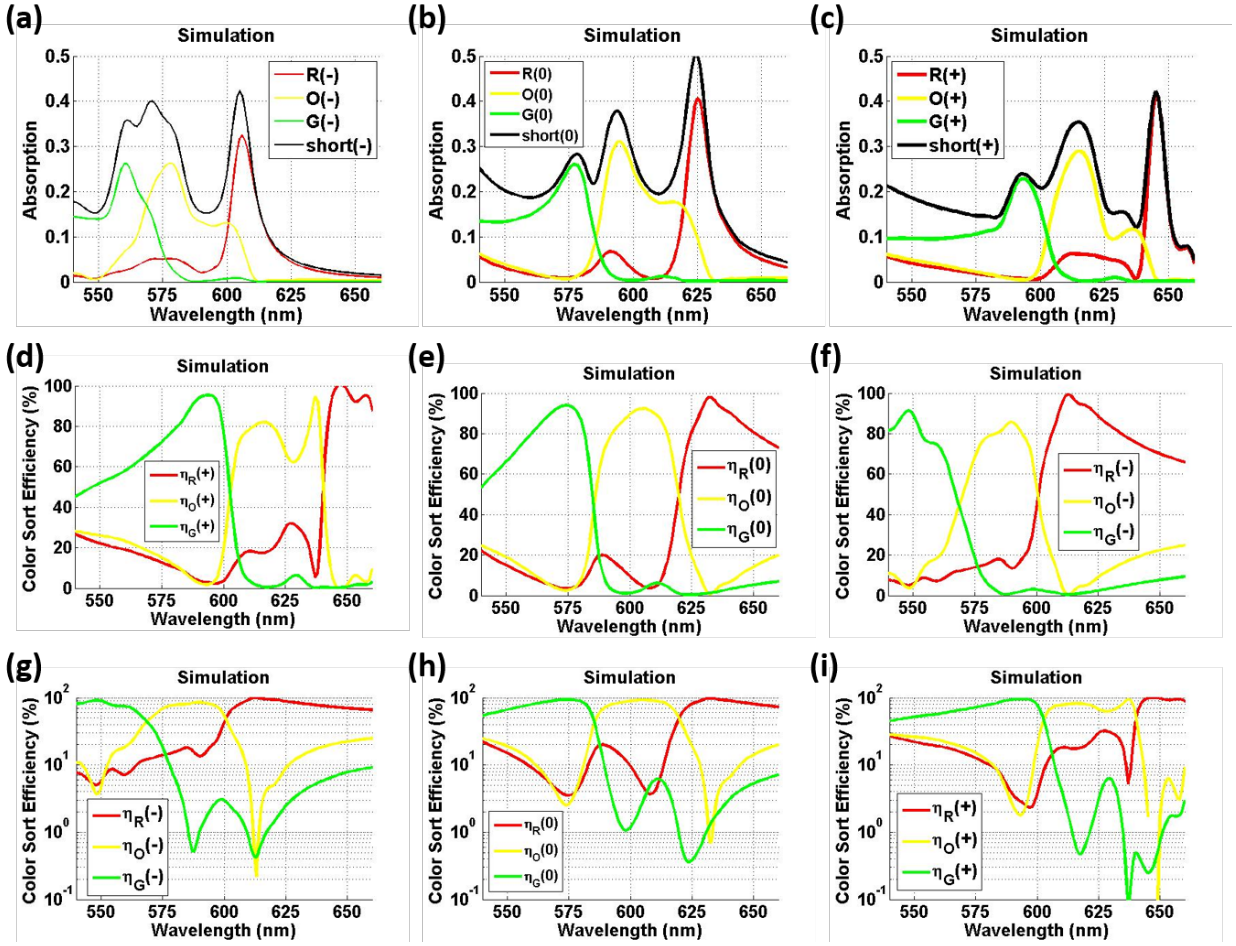

Supplementary Figure 2. Simulation of metasurface with varying diameter nanocylinders. (a-c) Simulated absorption spectra in [Green Yellow Red] color channels with nanocylinder diameters of (-) [131, 119, 97] nm, (0) [136, 124, 102] nm, (+) [131, 119, 97] nm, with the lattice constant fixed at 220 nm. (d-e) Corresponding color-sorting efficiency on linear (d-e) and logarithmic (g-i) scales.

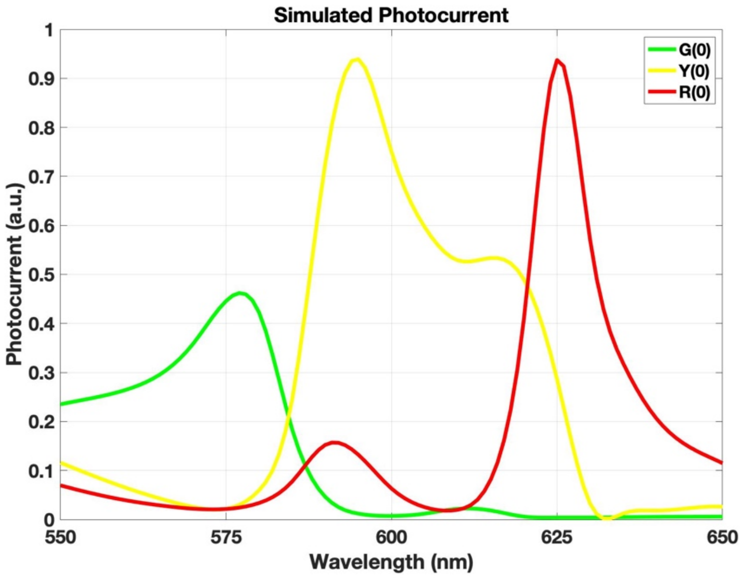

Supplementary Figure 3. Simulation of relative photocurrent in zero-scale AH devices. Conversion of optical energy into photocurrent was simulated by self-consistently solving Poisson's and the drift-diffusion equations using a commercial numerical solver (Lumerical DEVICE™). Results from electromagnetic simulations of absorption in the nanocylinders were used as the source fields for electrical simulations, with the geometry modeled by the finite element method. Photocurrent simulated as a function of illumination wavelength closely matches the simulated absorption spectra.

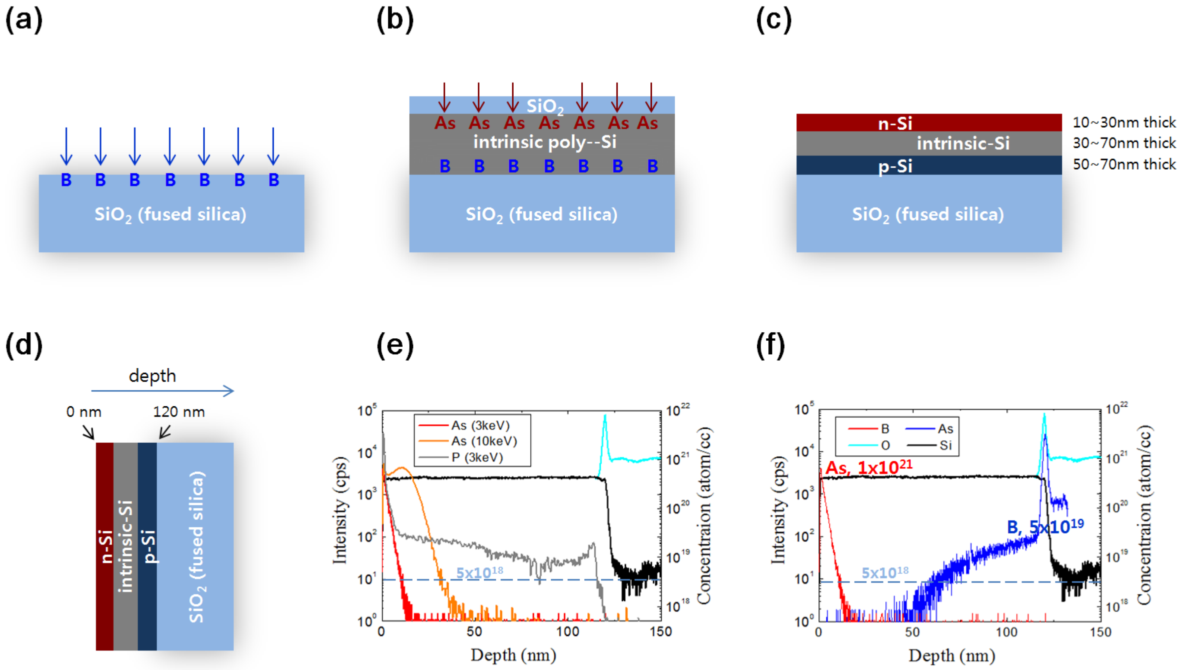

Supplementary Figure 4. Vertical shallow junction of PIN poly Si. (a) Boron ion implantation on fused silica substrate, implantation energy of 3keV, dose of $2x10^{16}cm^{-2}$. **(b)** Successive processes of 130nm-thick poly-Si deposition, 50nm-thick SiO_2 coating, and arsenic ion implantation. (ion energy of 3~10keV, dose of $1x10^{16}cm^{-2}$) **(c)** PIN Si formation by RTP activation for 10seconds at 1000°C, afterwards removing SiO_2 capping layer. **(d)** Schematic illustration of PIN Si. **(e)** The diffusion depths of n-type dopants, phosphorous and arsenic, into poly-Si, analyzed by secondary ion mass spectroscopy (SIMS). **(f)** Depth profile of PIN Si, having the thickness of 10nm, 50nm, 70nm for n-type, intrinsic, p-type Si respectively, analyzed by SIMS.

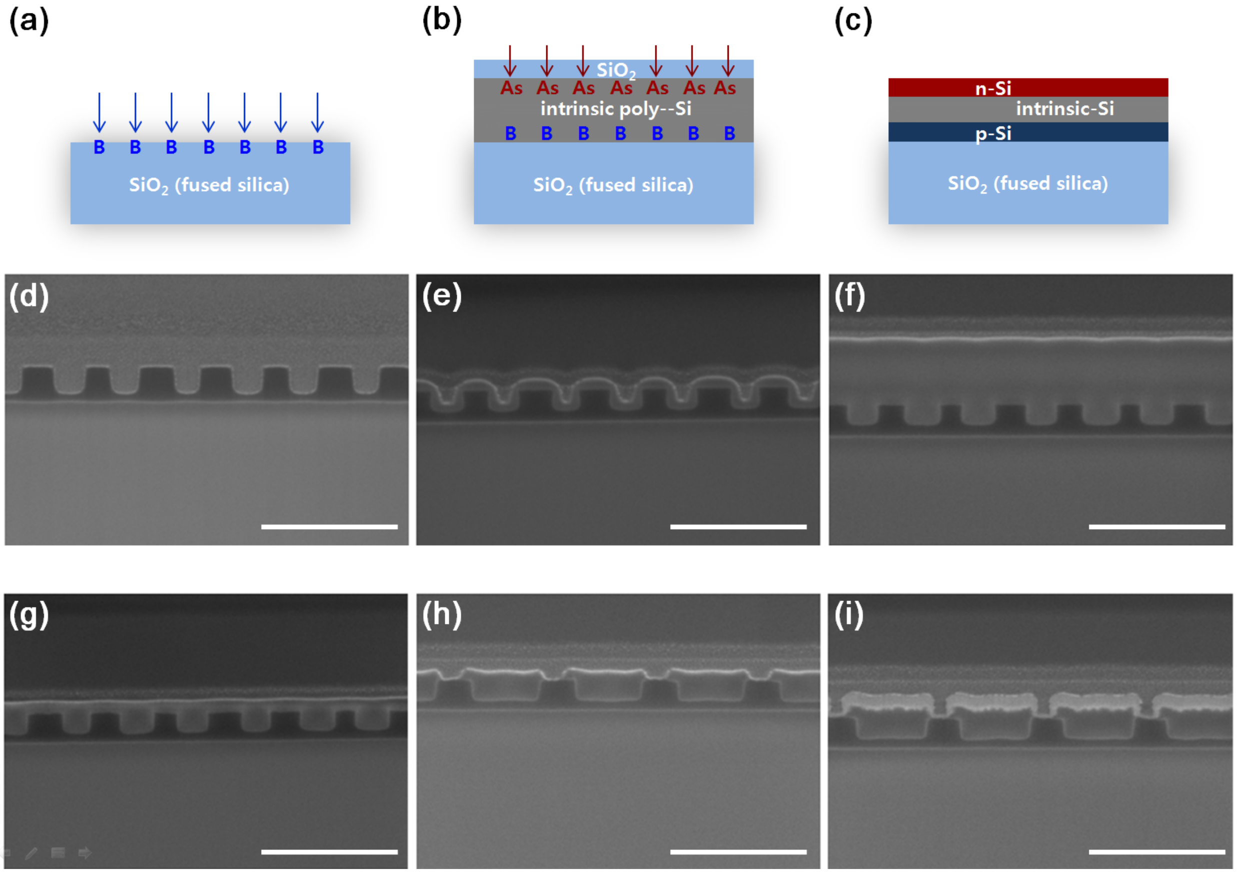

Supplementary Figure 5. Fabrication process for the AH PIN silicon metasurface. (a-c) Intrinsic silicon is doped n-type and p-type over a 130 nm height to create a shallow p-i-n junction. (d) Nano patterning of PIN poly-Si rods with vertical side slope. (e) Depositing 50nm-thick SiO_2, following the trimming process of protrusion edges by Ar plasma treatment. (f) Void-free nano gap filling and surface planarization. (g) By etching back, 30~50nm thickness of SiO_2 is left over upper regions of nano rod arrays. (h) Nano hole formation (i) ITO electrode deposition for the contact of ITO layer and n-Si cylinders. The white scale bar corresponds to 500 nm in (d-i).

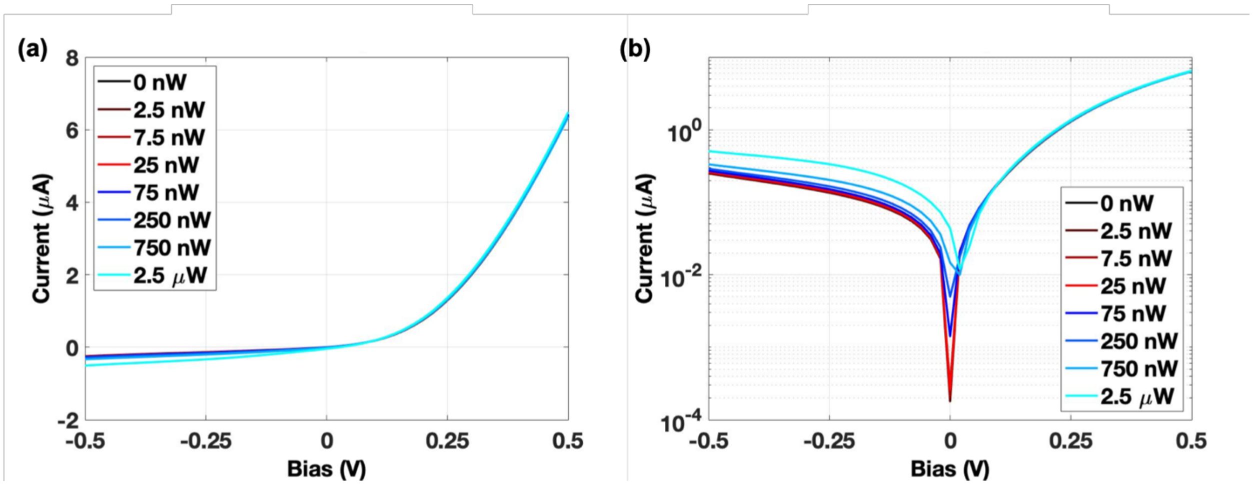

Supplementary Figure 6. Current-voltage relationship for representative AH device under illumination. (a) Linear and (b) logarithmic IV curves with illumination power as a parameter for a red-absorbing AH device at an illumination wavelength of 610 nm.

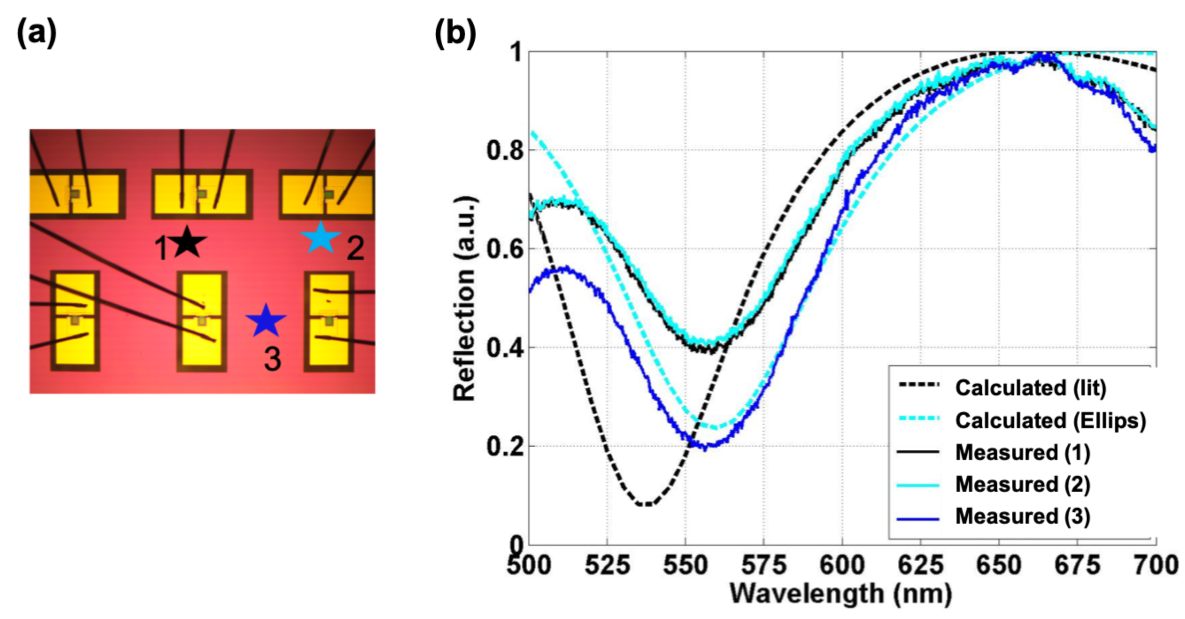

Supplementary Figure 7. Reflection spectra of control PIN silicon. (a) Spatial locations of measured reflection spectra on the control PIN silicon. (b). Corresponding measured reflection spectra, as well as calculated reflection spectra based on literature values of silicon optical constants and measured values via ellipsometry.

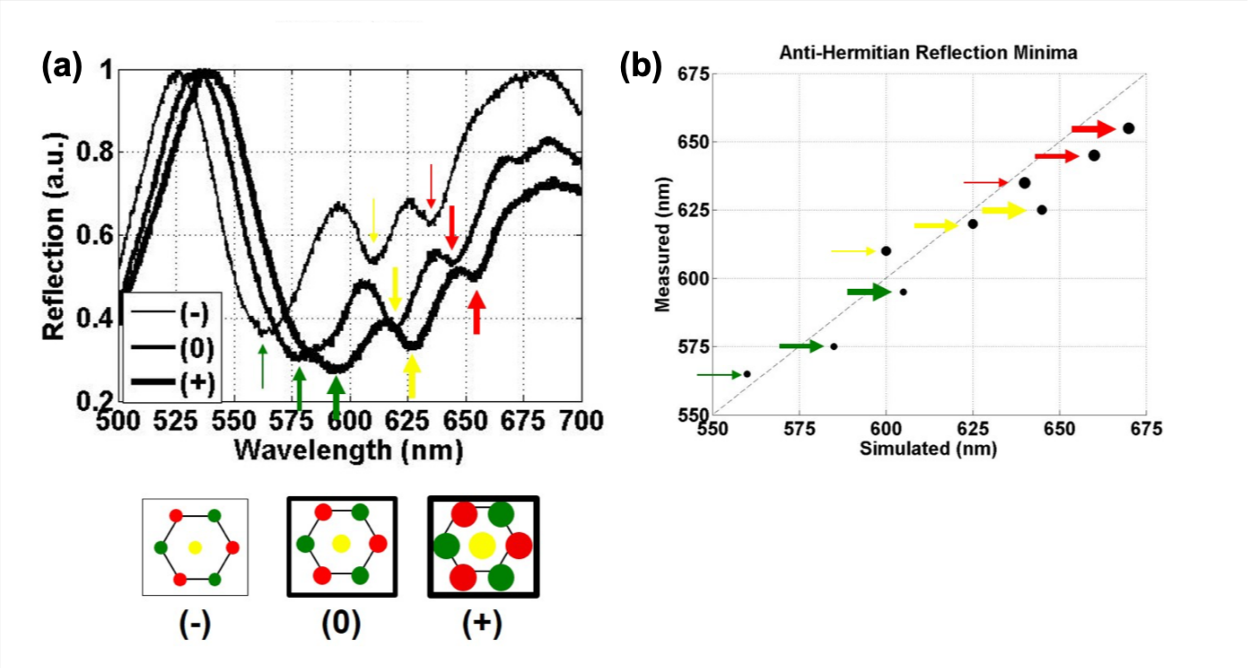

Supplementary Figure 8. Reflection spectra of AH metasurfaces with different scale factors. (a) Measured reflection as a function of wavelength for AH metasurfaces, with nanocylinder diameters of (-) [131, 119, 97] nm, (0) [136, 124, 102] nm, (+) [141, 129, 107] nm, with the center-to-center separation distance between nanocylinders fixed at 220 nm. For each metasurface, three reflection minima are clearly observed, associated with the green, yellow, and red color channels. Each minima red-shifts as the scale factor increases, consistent with expectations based on numerical design. (b) Measured reflection minima versus simulated reflection minima.

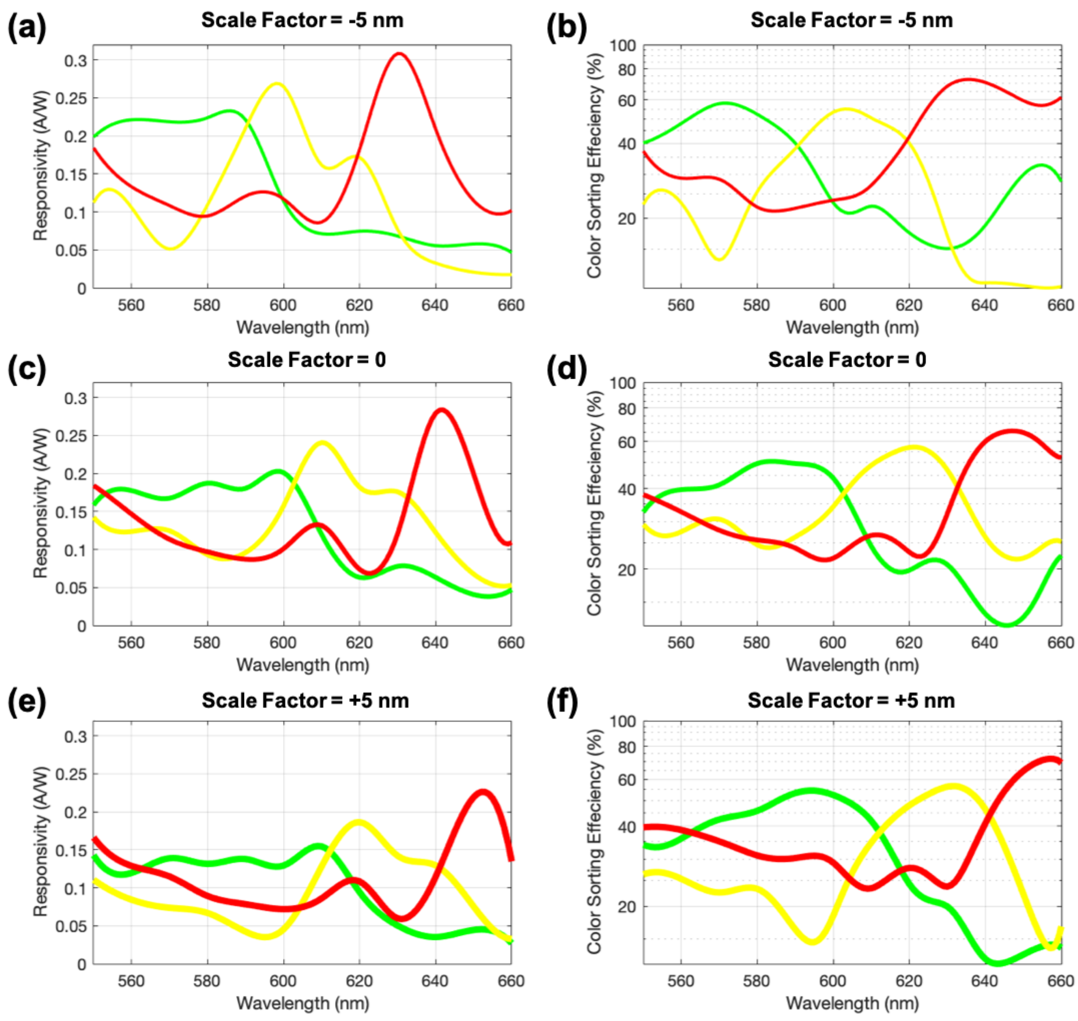

Supplementary Figure 9. Measured responsivity and color sorting efficiency of AH PIN silicon metasurfaces with different scale factors. Measured (a,c,e) responsivity and (b,d,f) color sorting efficiency, using a high NA objective, of AH metasurfaces with designed nanocylinder diameters of (a,b) [141, 129, 107] nm, (c,d) [136, 124, 102] nm, (e,f) [131, 119, 97] nm, with the lattice constant fixed at 220 nm.

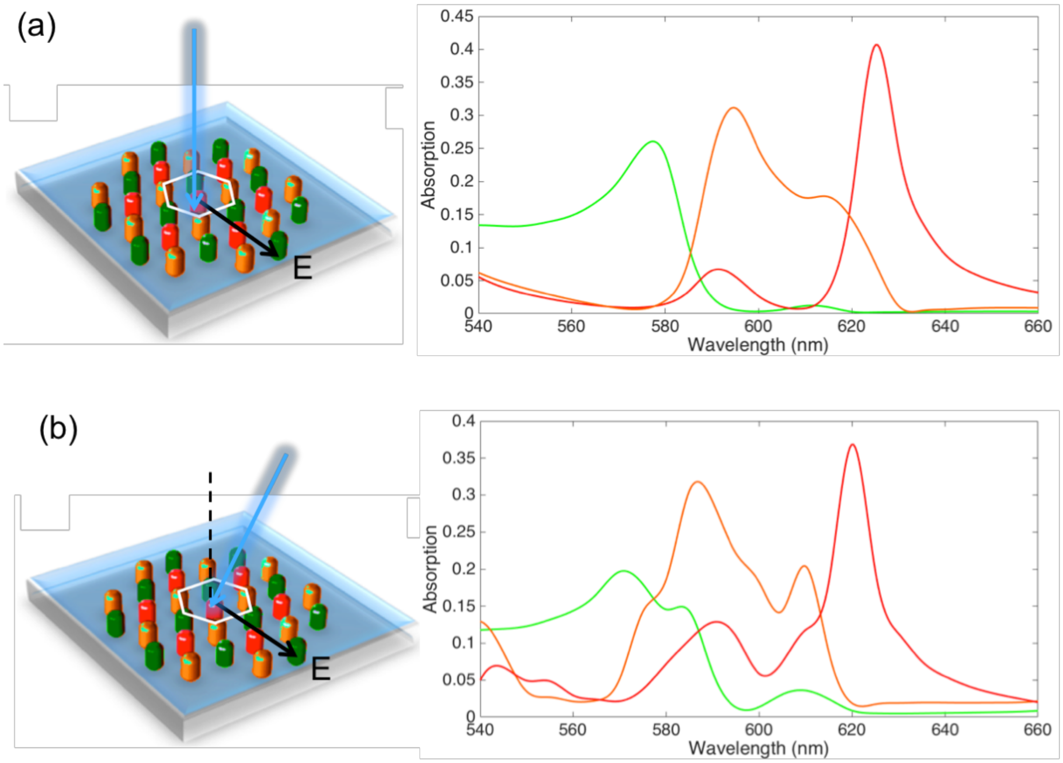

Supplementary Figure 10. Dependence of the device's performance on incident angle and polarization. (a) Simulated absorption spectra inside three kinds of cylinders under normal incidence. (b) Simulated absorption spectrum inside three kinds of cylinders under TE excitation at 30° incident angle.

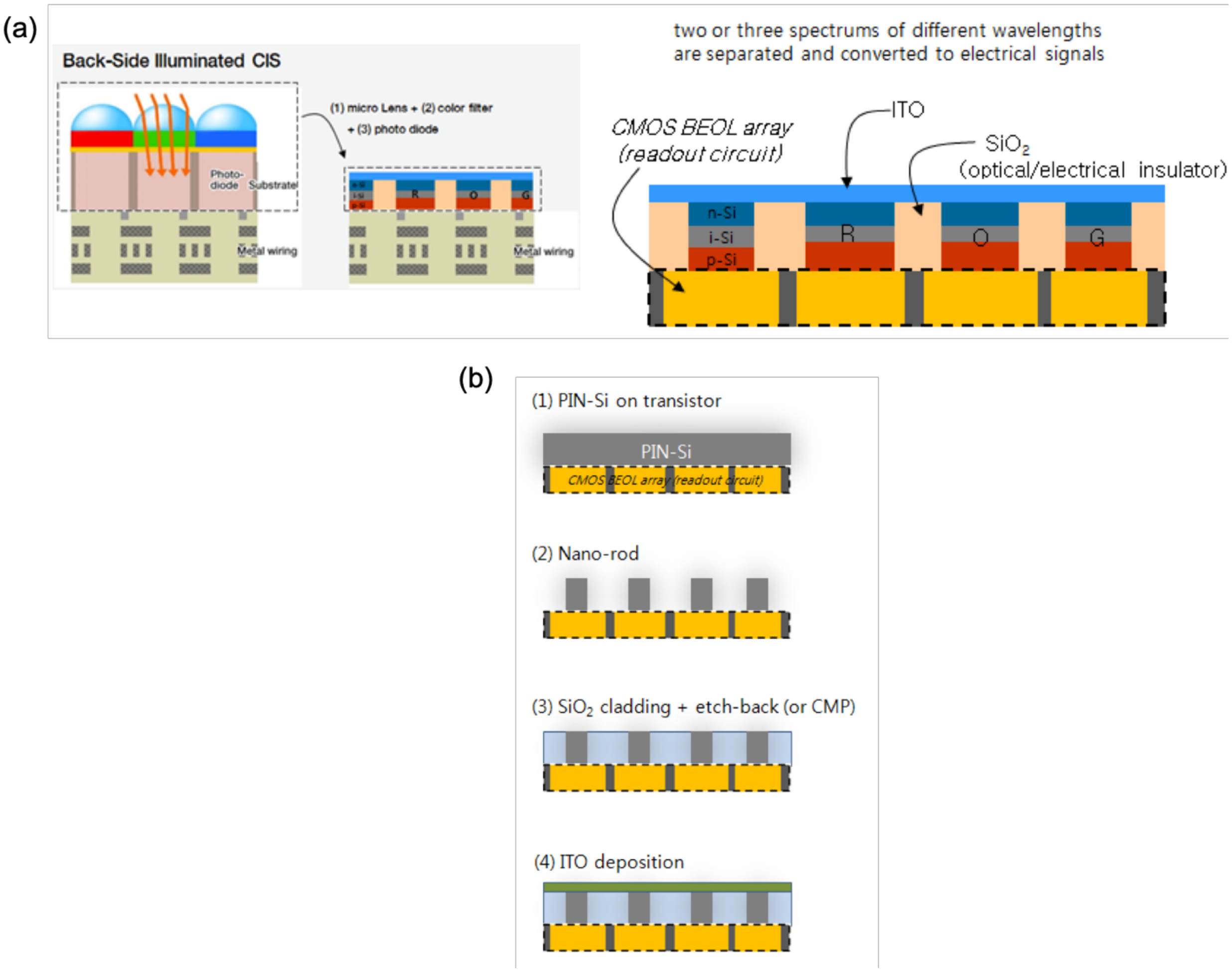

Supplementary Figure 11. Fabrication process including CMOS readout circuit array. (a) When the p-i-n nanocylinders of three different sizes are connected to the CMOS readout circuit array, then the three current signals are extracted from a single sample. The fabrication of CMOS readout circuit array is a publicly known semiconductor process. (b) Briefly, this process may be summarized by the following steps. Firstly, intrinsic silicon is doped n-type and p-type over a 130 nm height to create a shallow p-i-n junction, including implantation process of boron ions on the surface of CMOS readout circuits. Secondly, nanocylinders are formed by a plasma etching method of polycrystalline silicon. Thirdly, a low index material, SiO2 (n=1.45 at wavelength of 550 nm), is used to fill volume surrounding the nanocylinders. Lastly, a thin film of indium tin oxide (ITO) is coated as a common electrode.

Supplementary Table 1. Process parameters for achieving shallow junction p-i-n diode with variable p-type, intrinsic, and n-type silicon layer thicknesses.

Sample	Si thickness			Arsenic, implantation	Boron, RTP duration	Substrate
	N-type Si	Intrinsic Si	P-type Si			
i-Si 30	30nm	30nm	70nm	10keV	30 seconds	Fused silica
i-Si 50	10nm	50nm	70nm	3keV	30 seconds	Fused silica
i-Si 70	10nm	70nm	50nm	3keV	10 seconds	Fused silica

Supplementary Note 1

Optical resonances supported by silicon nanoparticles are typically broad with low quality factors, leading to detrimental optical crosstalk characterized by significant spectral overlap between different color channels. Here we show that by carefully tuning the separation distance between nanocylinders in the hexagonal metasurface, the couplings can be engineered close to the AH condition, resulting in sharpened resonances and reduced optical crosstalk. To analyze the coupling phenomena, we write the coupled equations that describes the silicon nanocylinder lattice under a normal illumination of light as[15]

$$\begin{pmatrix} -\omega + \omega_R - i\gamma_R & \kappa_{RY} & \kappa_{RG} \\ \kappa_{YR} & -\omega + \omega_Y - i\gamma_Y & \kappa_{YG} \\ \kappa_{GR} & \kappa_{GY} & -\omega + \omega_G - i\gamma_G \end{pmatrix} \begin{pmatrix} A_R \\ A_Y \\ A_G \end{pmatrix} = \begin{pmatrix} g_R \\ g_Y \\ g_G \end{pmatrix} E_0 \qquad (1)$$

In Eq. 1, ω_i and γ_i represents the resonance frequency and dissipation of nanocylinders of color i, κ_{ij} is the coupling coefficient between nanocylinders of color i and j, and g_i is the coupling constant between the incident field and the nanocylinder. Under a plane wave illumination of amplitude E_0 and frequency ω, resonant modes are excited in each type of nanocylinders with amplitude A_i.

The silicon nanocylinders can be viewed as quasi-bound states, which couple to each other via direct near-field coupling as well as indirect far-field coupling mediated by the radiation channels[6]. The real part of the coupling coefficient κ_{ij} consists of both the near-field coupling and the real part of the far-field coupling, while the imaginary part of κ_{ij} describes only the far-field coupling. When the separation distance between nanocylinders is smaller than the excitation wavelength, only zero-order diffraction can be induced, and the three types of nanocylinders will couple to the single radiation channel. In this coupling regime, and under the assumption that $\mathrm{Re}(\kappa_{jk}) = 0$, it has previously been derived that upon excitation at a resonance frequency ω_i, the amplitude in all other types of nanoresonators are suppressed to $A_{j\neq i} = 0$.[4] This indicates that, under normal illumination of the metasurface, multiple modes are simultaneously excited in different types of nanocylinders, which interfere constructively at one type of nanocylinders and destructively at all the others, leading to a sharpened resonance and increased quality factor.

Supplementary Note 2

It should be noted that the blue part of the visible spectrum was intentionally ignored in this work because the absorption of silicon increases exponentially with decreasing wavelength. The large absorption of blue light relative to green or red leads to a significant imbalance in the amount of light absorbed across the visible spectrum. Including blue wavelengths in an AH metasurface architecture likely requires multiple absorbing planes, the analysis of which may be the subject of future research.
